# Integer programming models and branch-and-cut approaches to generalized {0,1,2}-survivable network design problems

**DOI:** 10.1007/s10589-016-9836-y

**Published:** 2016-03-02

**Authors:** Markus Leitner

**Affiliations:** Department of Statistics and Operations Research, Faculty of Business, Economics and Statistics, University of Vienna, Vienna, Austria

**Keywords:** Generalized network design, Survivability, Biconnectivity, Branch-and-cut, Mixed integer linear programming

## Abstract

In this article, we introduce the Generalized $\{0,1,2\}$-Survivable Network Design Problem ($\{0,1,2\}$-GSNDP) which has applications in the design of backbone networks. Different mixed integer linear programming formulations are derived by combining previous results obtained for the related $\{0,1,2\}$-GSNDP and Generalized Network Design Problems. An extensive computational study comparing the correspondingly developed branch-and-cut approaches shows clear advantages for two particular variants. Additional insights into individual advantages and disadvantages of the developed algorithms for different instance characteristics are given.

## Introduction

The optimal design of (telecommunication) networks has been the topic of numerous scientific articles and a variety of different (classes of) combinatorial optimization problems arising in that domain have been studied in detail. *Generalized Network Design Problems (GNDPs)* are one particular class among these that are motivated from the design of backbone networks, see, e.g. [3, 4, 10, 22] and the references therein. In GNDPs we are given an undirected graph $G=(V,E)$ with nonnegative edge weights $c_e, \forall e\in E$, in which the set of nodes *V* is partitioned into *k* disjoint clusters $V_i, 1\le i\le k$. Thereby, each node represents a possibility to connect a local network to a backbone network and all nodes within the same cluster belong to the same local network. Usually, the goal is to identify a minimum-cost backbone network such that precisely one node from each local network is connected to it. Depending on the additional constraints on the structure of the backbone network one obtains different GNDPs such as the Generalized Minimum Spanning Tree Problem (GMSTP), see, e.g. [5, 6, 9, 13, 19, 21, 23, 24], or the Generalized Traveling Salesman Problem, cf. [7, 11, 16, 27].

To ensure connectivity of the backbone network even after the outage of a single link, Huygens [15] introduced the generalized minimum edge biconnected network design problem (GMEBCNP) and studied possibilities to model this problem as an integer linear program (ILP). The GMEBCNP as well as a variant in which connectivity is maintained even after a single node failure have also been studied in [12, 14] where mainly (meta-) heuristic approaches are proposed. While the issue of survivability has received only little attention for the case of generalized network design, a huge amount of literature is available for “classical” survivable network design (SND), see, e.g. [20]. One particular subclass that is of major relevance for what follows are the so-called {0,1,2}-SND problems where a value associated to each node specifies the required level of redundancy, see, e.g. [2].

In this work, we generalize the latter problem to the case of generalized network design by introducing the *Generalized {0,1,2}-Survivable Network Design Problem ({0,1,2}-GSNDP)*. To formally define the problem, let $G=(V,E)$ be an undirected graph with nonnegative weights $c_e\ge 0$ associated to edges $e\in E$ whose node set is partitioned into *k* disjoint clusters $V_i, i=1, \ldots , k$. Let furthermore, $\rho _i\in \{0,1,2\}, i=1,\ldots , k$, denote the connectivity requirements of cluster *i*. Thereby, *mandatory clusters* with $\rho _i\in \{1,2\}$ need to be connected in any feasible solution while all remaining ones (with $\rho _i=0$) are *optional clusters*. As in classical survivable network design problems, *redundant clusters*$V_i$ with $\rho _i=2$ need to be connected redundantly, i.e., they need to remain connected (to all other redundant clusters) after a single node or edge failure.

**Fig. 1:**
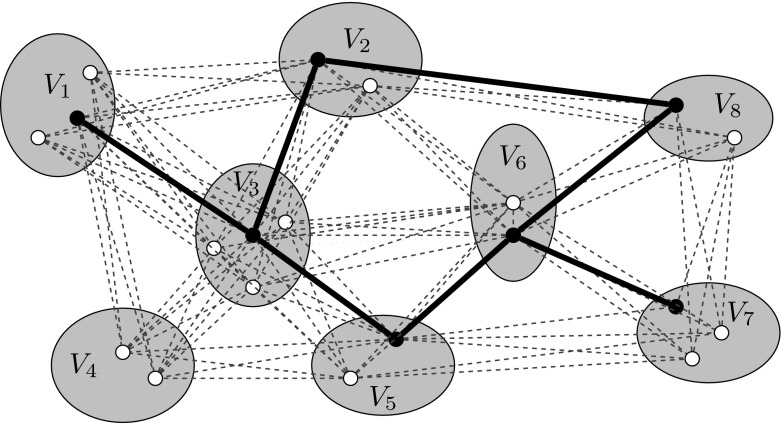
An instance and a feasible solution of an instance to the {0,1,2}-GSNDP for $\rho _i=2, i\in \{2,5,8\}, \rho _i=1, i\in \{1,6,7\}$, and $\rho _i=0, i\in \{3,4\}$. *Dotted* edges are not included in the solution

A feasible solution $G'=(V',E')$ to an instance of the {0,1,2}-GSNDP is a subgraph of *G* that contains precisely one node from each mandatory cluster and at most one node from each optional cluster. Furthermore, for each pair of clusters $V_i$ and $V_j (i,j\in \{1, \ldots , k\}, i\ne j$) solution $G'$ has to contain at least $\rho _{ij}=\min \{\rho _i, \rho _j\}$ vertex disjoint paths connecting the nodes selected in these clusters. Thus, two redundant clusters $V_i$ and $V_j$ remain connected after a single node or edge failure (unless the selected node in either $V_i$ or $V_j$ is the one failing). The objective is to identify a solution $G^*=(V^*,E^*)$ yielding overall minimum edge costs $\sum _{e\in E^*} c_e$. Figure 1 shows an example instance of the {0,1,2}-GSNDP together with a solution to this instance.

*Scientific contribution and outline of the paper* The main contributions of this article are the introduction of the new problem with applications in backbone network design and the development of (mixed) integer linear programming ((M)ILP) formulations of polynomial and exponential size. As these formulations heavily rely on a recent result by Chimani et al. [2], we recall this result and discuss its consequences at the end of this section after summarizing necessary notation. Compact MILP formulations involving a polynomial number of variables and constraints (w.r.t. to the size of the input graph) based on multi-commodity flows are introduced in Sect. 2.1. ILP formulations with an exponential number of (efficiently separable) constraints are proposed in Sect. 2.2. In addition we analyze possibilities to formulate the problem by using an exponential number of path and cycle variables in Sect. 2.3. Branch-and-cut approaches for the formulations from Sects. 2.1 and 2.2 are developed and compared from a computational perspective in Sect. 3 using well known benchmark instances for GNDPs. Finally, conclusions are drawn in Sect. 4. We also note, that all formulations described in the following are easily modified to the edge-disjoint variant of the problem. They can also be directly used for the important special case when all clusters need to be connected redundantly, i.e., when $\rho _i=2, 1\le i\le k$.

*Notation and assumptions* In the following, $C=\{1,\ldots , k\}$ is used to denote the set of clusters and $C_l=\{i\in C\mid \rho _i=l\}, l\in \{0,1,2\}$, to easily distinguish between the different cluster classes. Throughout this article we will also assume that there exist at least two type-2 clusters, i.e., $|C_2|\ge 2$. For $C'\subseteq C$, we will also use $V(C')=\bigcup _{i\in C'} V_i$ to denote all nodes of cluster subset $C'$. In the formulations in Sect. 2, we will also make use of arc set $A=\{(u,v)\mid \{u,v\}\in E\}$ obtained by bi-directing edge set *E* and assume that the given cost function is appropriately defined on it as well, i.e., $c_{uv}=c_{vu}=c_e, \forall e=\{u,v\}\in E$. For node sets $S,S'\subset V$, common notation for cutsets $\delta ^+(S)=\{(u,v)\in A\mid u\in S, v\notin S\}, \delta ^-(S)=\{(u,v)\in A\mid u\notin S, v\in S\}$, and $\delta (S,S')=\{(u,v)\in A\mid u\in S, v\in S'\}$ will be used. Notation $A(w)=\delta ^+(\{w\})\cup \delta ^-(\{w\})$ will be used to refer to the set of arcs adjacent to node $w\in V$. Finally, for a set of variables $\omega$ defined on set $\Omega$ and subset $\Omega '\subseteq \Omega$, we will use notation $\omega [\Omega ']=\sum _{i\in \Omega '} \omega _i$.

*Orientation of 2-node connected graphs* All MILP formulation introduced in the following section exploit the following recent orientation result for 2-node connected graphs by Chimani et al. [2].

### **Theorem 1**

(Chimani et al. [2]) An undirected graph $G'=(V',E')$ is 2-node-connected if and only if for an arbitrary chosen root node $s\in V'$ there exists an orientation $\hat{G}$ such that the in-degree of the root node is exactly 1 and for each node $v\in V'\setminus \{s\}, \hat{G}$ contains a directed path from *s* to *v* and a directed path from *v* to *s* which are node-disjoint except for *s* and *v*.

Chimani et al. [2] used their result to derived strong ILP formulations and effective branch-and-cut approaches for the {0,1,2}-SNDP. For the $\{0,1,2\}$-GSNDP studied in this article, their result implies that each solution can be oriented based on an arbitrarily chosen “root cluster” $r\in C_2$ as follows: (i) There exists a directed path $P_i\subset A$ from the node chosen in $V_r$ to a node selected in any other mandatory cluster $i\in C_1\cup C_2$; (ii) There exists a directed path $P_i'\subset A$ from a node selected in cluster $i\in C_2$ to the chosen node in $V_r$ that is node disjoint with $P_i$ except for its start and end node in $V_i$ and $V_r$, respectively.

This characterization has two important consequences for MILP formulations such as the ones introduced in the next section: (i) Instead of the need to consider paths between all pairs of (mandatory) clusters, it is sufficient to consider paths from the root cluster to all other mandatory clusters and from all redundant clusters to the root cluster. (ii) It allows to derive directed formulations which have been shown to be theoretically stronger than undirected ones for many related problems. More specifically, the directed formulations derived by Chimani et al. [2] were shown to theoretically dominate a previously existing undirected one and in fact their proof techniques could be used directly to derive similar results for the $\{0,1,2\}$-GSNDP. Thus in the following we refrain from giving the details which would require to additionally introduce undirected counterparts of our formulations.

## Integer programming formulations

In this section, we detail our directed (mixed) integer linear programming formulations for the {0,1,2}-GSNDP. All formulations will make use of decision variables $x_{uv}\in \{0,1\}, \forall (u,v)\in A$, indicating whether or not arc (*u*, *v*) is included in the (directed) solution and variables $z_i\in \{0,1\}, \forall i\in V$, which denote membership of node *i* in the solution subgraph.

### Flow formulations

Next, we describe multi-commodity formulations for the {0,1,2}-GSNDP that differ in the number of involved flow variables and their interpretation.

A first and somewhat natural flow formulation $(\mathrm {F}_{sv})$ one may think of would be based on establishing flows between relevant pairs of selected nodes. Thus, flow variables $f_{uv}^{st}, \forall s\in V_r, \forall t\in V(C_1\cup C_2\setminus \{r\}), \forall (u,v)\in A$, would indicate the amount of flow sent from node $s\in V_r$ to node *t* from a mandatory cluster. Furthermore, flow variables $g_{uv}^{ts}, \forall t\in V(C_2\setminus \{r\}), \forall s\in V_r, \forall (u,v)\in A$, would indicate the flow sent back from a node *t* that requires a redundant connection (if selected) to a node *s* from the root cluster *r*. In addition, binary variables $w_{sv}, \forall s\in V_r, \forall v\in V(C_1\cup C_2\setminus \{r\})$, would be used. The latter would be equal to one if and only if both *s* and *v* are included in the solution (i.e., $w_{sv}=z_s z_v$) and thus indicate whether or not a connection needs to be installed between them.

It is well known, however, that using standard techniques to linearize equations $w_{sv}=z_s z_v$ one obtains an extremely weak formulation. In fact, as will be detailed in Sect. 2.2, the linear programming (LP) relaxation of a resulting formulation will be close to zero in almost all cases. To summarize, one obtains a theoretically weak formulation with a quite huge number of variables. Thus, we refrain from giving further details. In Sect. 2.2 we will, however, introduce an analogous formulation but which avoids the use of flow variables through exponentially many constraints.

Our next model given by (1)–(13) to which we will refer to as $(\mathrm {F}_{u})$ significantly reduces the number of necessary flow variables and eliminates the “quadratic” variables $w_{sv}$ of above variant. Formulation $(\mathrm {F}_u)$ is based on flow variables $f^t_{uv}, \forall t\in V(C_1\cup C_2\setminus \{r\}), \forall (u,v)\in A$, indicating the amount of flow sent from the node selected in $V_r$ to node *t* selected in a mandatory cluster along arc (*u*, *v*). Similarly, flow variables $g^t_{uv}, \forall t\in V(C_2\setminus \{r\}), \forall (u,v)\in A$, denote the flow along arc (*u*, *v*) of the required backward path from node *t* chosen in a redundant cluster to the node selected in $V_r$

1 $$\begin{aligned} \min \,&\sum _{(u,v)\in A} c_{uv} x_{uv} \end{aligned}$$

2 $$\begin{aligned} \text{ s.t. }\,&z[V_i]=1&i\in C_1\cup C_2 \end{aligned}$$

3 $$\begin{aligned}&z[V_i]\le 1&i\in C_0 \end{aligned}$$

4 $$\begin{aligned}&x[\delta ^-(u)] = z_u&u\in V_r \end{aligned}$$

5 $$\begin{aligned}&x_{uv}+x_{vu} \le z_v&v\in V,\ \{u,v\}\in E \end{aligned}$$

6 $$\begin{aligned}&f^{t}[\delta ^+(u)] - f^{t}[\delta ^-(u)] {\left\{ \begin{array}{ll} \ge z_u + z_t - 1 &{} \quad \text{ if } u\in V_r \\ = -z_u &{} \quad \text{ if } u\in V_t \\ = 0 &{} \quad \text{ otherwise } \end{array}\right. }&\nonumber \\&\quad \quad \quad \quad \quad \quad \quad \quad \quad \quad \quad \quad \quad t\in V(C_1\cup C_2\setminus \{r\}),\ u\in V \end{aligned}$$

7 $$\begin{aligned}&g^{t}[\delta ^+(u)] - g^{t}[\delta ^-(u)] {\left\{ \begin{array}{ll} = z_u &{} \quad \text{ if } u\in V_t \\ \le 1-z_u-z_t &{} \quad \text{ if } u\in V_r \\ 0 &{} \quad \text{ otherwise } \end{array}\right. }&\nonumber \\&\quad \quad \quad \quad \quad \quad \quad \quad \quad \quad \quad \quad \quad t\in V(C_2\setminus \{r\}),\ u\in V \end{aligned}$$

8 $$\begin{aligned}&f^{t}[\delta ^-(u)] + g^{t}[\delta ^-(u)] \le z_t&t\in V(C_2\setminus \{r\}),\ u\in V \end{aligned}$$

9 $$\begin{aligned}&f^{t}_{uv}\le x_{uv}&t\in V(C_1),\ (u,v)\in A \end{aligned}$$

10 $$\begin{aligned}&f^t_{uv} + g^{t}_{uv}\le x_{uv}&t\in V(C_2\setminus \{r\}),\ (u,v)\in A \end{aligned}$$

11 $$\begin{aligned}&f^t_{uv}\ge 0&t\in V(C_1\cup C_2\setminus \{r\}),\ (u,v)\in A \end{aligned}$$

12 $$\begin{aligned}&g^t_{uv}\ge 0&t\in V(C_2\setminus \{r\}),\ (u,v)\in A\end{aligned}$$

13 $$\begin{aligned}&(\mathbf{x},\mathbf{z})\in \{0,1\}^{|A|+|V|} \end{aligned}$$

The objective function (1) minimizes the installation costs of all arcs included in the (directed) solution, while constraints (2) and (3) ensure that exactly (at most) one node is selected within each mandatory (optional) cluster. Equation (4) are indegree constraints for all nodes from the root cluster that are valid since the selected root node must have indegree equal to one, cf. aforementioned result by Chimani et al. [2]. Inequalities (5) ensure that at most one among each pair of oppositely directed arcs can be selected and that only arcs (*u*, *v*) for which nodes *u* and *v* are selected as well may be used. Constraints (6) ensure that exactly one unit of flow $f^t, t\in V(C_1\cup C_2\setminus \{r\})$, is consumed by node *t* (if selected) and that the corresponding flow can only be sent out from a node $u\in V_r$. Using the linking constraints (5) and (9) we observe that indeed flow $f^t$ can only be produced at the node selected within $V_r$. Thus, one unit of flow will be sent from the node selected in $V_r$ to each selected node contained in a mandatory cluster. By similar arguments, one can conclude that the required backward paths from selected nodes within redundant clusters will be established due to constraints (7) and the two sets of linking constraints. Inequalities (8) ensure node-disjointness of flows $f^t$ and $g^t$ for each selected node $t\in V(C_2\setminus \{r\})$ while (9) and (10) link flow and arc design variables. Finally, notice that $(\mathrm {F}_u)$ does not contain unnecessary flow variables $f^t_{us}, s\in V_r, (u,s)\in A$, and $g^t_{ut}, (u,t)\in A$, which are however included in above formulation to simplify notation.

Pop [22] observed that the GMSTP can be modeled by using variables describing inter-cluster (which he called “global”) connections. Using a classical spanning-tree model for describing the set of feasible “global” solutions together with additional variables and constraints ensuring that the cheapest “local” connections corresponding to a particular global solution is chosen, he proposed a new MILP model for the GMSTP [22]. This idea has turned out to also allow to derive quite effective heuristic approaches to different GNDPs, see, e.g. [12–14]. Recently, it has also been used to derive an efficient branch-and-cut approach for the GMSTP with hop constraints by the current author [17].

Next, we show how to exploit this concept in order to derive an additional multi-commodity flow formulation for the {0,1,2}-GSNDP with a significantly smaller number of flow variables than the two formulations described above. Thereby, arc set $A_\mathrm{C}=\{(i,j)\mid \exists (u,v)\in A, u\in V_i, v\in V_j\}$ is used that contains an arc between a pair of clusters whenever the there exists at least one edge between two nodes from the corresponding clusters in the original graph *G*. In formulation (14)–(20), paths from the root cluster to each other required cluster *k* are realized by using flow variables $f_{ij}^k, \forall k\in C_1\cup C_2\setminus \{r\}, \forall (i,j)\in A_\mathrm{C}$, while paths from each redundant cluster *k* back to the root cluster are realized using flow variables $g_{ij}^k, \forall k\in C_2\setminus \{r\}, \forall (i,j)\in A_\mathrm{C}$. As before both kinds of variables indicate the amount of flow of a particular type on each arc. Indicating that flows are actually sent between clusters rather than between nodes, $(\mathrm {CF})$ will be used to refer to model (14)–(20) in the following.

Note that $(\mathrm {CF})$ also uses notation $\delta _\mathrm {c}^+(i)$ (and $\delta _\mathrm {c}^-(i)$) to refer to the set of outgoing (incoming) inter-cluster arcs from $A_\mathrm{C}$ that are adjacent to cluster *i*.

14 $$\begin{aligned} \min \,&\sum _{(u,v)\in A} c_{uv} x_{uv} \end{aligned}$$

15 $$\begin{aligned} \text{ s.t. }\,&(2)-(5) \nonumber \\&f^{k}[\delta _\mathrm {c}^+(i)] - f^{k}[\delta _\mathrm {c}^-(i)] = {\left\{ \begin{array}{ll} 1 &{} \quad \text{ if } i=r \\ -1 &{} \quad \text{ if } i=k \\ 0 &{} \quad \text{ otherwise } \end{array}\right. }&\nonumber \\&k\in C_1\cup C_2\setminus \{r\},\ i\in C \end{aligned}$$

16 $$\begin{aligned}&g^{k}[\delta _\mathrm {c}^+(i)] - g^{k}[\delta _\mathrm {c}^-(i)] = {\left\{ \begin{array}{ll} 1 &{} \quad \text{ if } i=k \\ -1 &{} \quad \text{ if } i=r \\ 0 &{} \quad \text{ otherwise } \end{array}\right. }&\nonumber \\&k\in C_2\setminus \{r\},\ i\in C \end{aligned}$$

17 $$\begin{aligned}&f^{k}[\delta _\mathrm {c}^-(i)] + g^{k}[\delta _\mathrm {c}^-(i)] \le z[V_i]&k\in C_2\setminus \{r\},\ i\in C \end{aligned}$$

18 $$\begin{aligned}&0\le f^{k}_{ij}\le x[\delta (V_i,V_j)]&k\in C_2\setminus \{r\},\ (i,j)\in A_\mathrm{C} \end{aligned}$$

19 $$\begin{aligned}&0\le g^{k}_{ij}\le x[\delta (V_i,V_j)]&k\in C_2\setminus \{r\},\ (i,j)\in A_\mathrm{C} \end{aligned}$$

20 $$\begin{aligned}&(\mathbf{x},\mathbf{z})\in \{0,1\}^{|A|+|V|} \end{aligned}$$

Flow conservation constraints (15) and (16) together with disjointness constraints (17) model the necessary (disjoint) flows on the inter-cluster level while linking constraints (18) and (19) ensure that for each used inter-cluster connection at least one original arc is chosen as well.

A valid formulation is obtained together with previously discussed constraints (2)–(5) that ensure that precisely one node is selected within each cluster to (or through) which flow is sent and that only arcs adjacent to two selected nodes may be used.

*Aggregated flow formulations* We note that alternative—but usually theoretically significantly weaker—formulations could be obtained by certain aggregations of flow variables in the formulations above. One such option is to consider only a single commodity of flow variables for all type-1 clusters (nodes in such clusters, respectively). Thus, $|C_1|$ units of such flow will be sent out from (the node selected in) the root cluster while one unit is consumed by each (node selected in a) type-1 cluster. It is, however, well known that the additional coefficient $|C_1|$ in the constraints linking flow and arc design variables will result in weak LP relaxation bounds and thus the resulting formulations typically do not perform well from a computational perspective. In addition, this concept cannot be used for (nodes selected in) type-2 clusters as the information on the destination (source) of each commodity is crucial to ensure the existence of the two disjoint paths.

### Cut formulations

It is well known that branch-and-cut approaches based on formulations utilizing an exponential number of directed connectivity constraints often outperform approaches based on flow-based models. In this section we describe three such models that conceptually correspond to the flow formulations described above. Thus, we start with model $(\mathrm {C}_{sv})$ defined by (21)–(28) which uses the “quadratic” variables $w_{sv}$ introduced above for model $(\mathrm {F}_{sv})$; besides using the arc and node design variables $x_{uv}$ and $z_u$, respectively.

21 $$\begin{aligned}&\min \, \sum _{(u,v)\in A} c_{uv} x_{uv} \end{aligned}$$

22 $$\begin{aligned}&\text{ s.t. }\, (2)-(5) \nonumber \\&w_{sv}\le z_s \qquad s\in V_r,\ v\in V(C_1\cup C_2\setminus \{r\}) \end{aligned}$$

23 $$\begin{aligned}&w_{sv}\le z_v \qquad s\in V_r,\ v\in V(C_1\cup C_2\setminus \{r\}) \end{aligned}$$

24 $$\begin{aligned}&w_{sv} \ge z_s + z_v - 1 \qquad s\in V_r,\ v\in V(C_1\cup C_2\setminus \{r\}) \end{aligned}$$

25 $$\begin{aligned}&x[\delta ^-(S)]\ge w_{sv} \qquad S\subset V,\ s\in V_r\setminus S,\ v\in V(C_1\cup C_2\setminus \{r\})\cap S \end{aligned}$$

26 $$\begin{aligned}&x[\delta ^+(S)]\ge w_{sv} \qquad S\subset V,\ s\in V_r\setminus S,\ v\in V(C_2\setminus \{r\})\cap S \end{aligned}$$

27 $$\begin{aligned}&x[\delta ^-(S_1)\setminus A(u)] + x[\delta ^+(S_2)\setminus A(u)] \ge w_{sv} \qquad u\in V\setminus \{s,v\}, \nonumber \\&S_1, S_2\subset V,\ s\in V_r\setminus (S_1\cup S_2),\ v\in V(C_2\setminus \{r\})\cap S_1\cap S_2 \end{aligned}$$

28 $$\begin{aligned}&(\mathbf{w},\mathbf{x},\mathbf{z})\in \{0,1\}^{|V_r|\times |V(C_1\cup C_2\setminus \{r\})|+|A|+|V|} \end{aligned}$$

Constraints (2)–(5) have been discussed in Sect. 2.1. Constraints (22)–(24) are the linear inequalities stating that variable $w_{sv}$ is equal to one if and only if nodes *s* and *v* are part of the solution. Inequalities (25) are directed connectivity cuts that ensure the existence of a path from the selected node in $V_r$ to any other node from a mandatory cluster that is included in the solution. Similarly, directed connectivity constraints (26) enforce the required path from a chosen node that needs to be connected redundantly to the selected root node. Disjointness of the two paths associated to selected nodes from redundant clusters is guaranteed due to connectivity cuts (27) which have also been used by Chimani et al. [2]. The latter state, that a selected node $v\in V(C_2\setminus \{r\})$ must still be connected to a chosen node $s\in V_r$ (in one of the two directions) after removing an arbitrary node $u\in V\setminus \{s,v\}$ and all its adjacent arcs.

Theorem 2 shows that $(\mathrm {C}_{sv})$ is a quite weak formulation.

#### **Theorem 2**

If every mandatory cluster contains at least two nodes, then the optimal value of the LP relaxation of $(\mathrm {C}_{sv})$ does not exceed $\min \{\frac{c_{us}+c_{vs'}}{2}\mid \{(u,s),(v,s')\}\subseteq \delta ^-(V_r), s\ne s'\}$.

#### *Proof*

For each mandatory cluster $i\in C_1\cup C_2$, let $u_i$ and $v_i$ be two nodes in $V_i, u_i\ne v_i$. We first observe that assigning values of 0.5 to the corresponding node variables ($z_{u_i}=z_{v_i}=0.5, \forall i\in C_1\cup C_2$) values $w_{sv}=0, \forall s\in V_r, \forall v\in V(C_1\cup C_2\setminus \{r\})$, are feasible according to inequalities (22)–(24). Hence, only indegree constraints (4) of nodes from $V_r$ force nonzero values of arc variables. Let *s* and $s'$ be the two nodes from $V_r$ such that $z_s=z_{s'}=0.5$. Then, a feasible LP solution can be constructed by setting $x[\delta ^-(s)]=x[\delta ^-(s')]=0.5$. The theorem follows by choosing $s,s'\in V_r$ and arcs $(u,s),(v,s')\in \delta ^-(V_r)$ (with $x_{us}=x_{vs'}=0.5$) in a cost minimal way. $\square$

We note, that one can strengthen formulation $(\mathrm {C}_{sv})$ by equations

29 $$\begin{aligned}&\sum _{s\in V_r} \sum _{v\in V_i} w_{sv} = 1&i\in C_1\cup C_2\setminus \{r\} \end{aligned}$$

These equations ensure that each cut containing all nodes from the root cluster on its source (target) and all nodes from another mandatory (redundant) cluster on its target (source) is at least one. It is immediate that they are strengthening as, e.g. the solution described in the proof of Theorem 2 violates them.

Similar to $(\mathrm {F}_{u})$, model (30)–(34) denoted as $(\mathrm {C}_{u})$ avoids using variables indicating whether a pair of nodes is included in the solution but instead focuses on cuts between the root cluster and other selected nodes.

30 $$\begin{aligned} \min \,&\sum _{(u,v)\in A} c_{uv} x_{uv} \end{aligned}$$

31 $$\begin{aligned} \text{ s.t. }\,&(2)-(5) \nonumber \\&x[\delta ^-(S)]\ge z_u \qquad S\subset V\setminus V_r,\ u\in V(C_1\cup C_2\setminus \{r\})\cap S \end{aligned}$$

32 $$\begin{aligned}&x[\delta ^+(S)]\ge z_u \qquad S\subset V\setminus V_r,\ u\in V(C_2\setminus \{r\})\cap S \end{aligned}$$

33 $$\begin{aligned}&x[\delta ^-(S_1)\setminus A(w)] + x[\delta ^+(S_2)\setminus A(w)] \ge z_u \qquad w\in V\setminus (V_r\cup \{u\}),\nonumber \\&S_1, S_2\subset V\setminus V_r,\ u\in V(C_2\setminus \{r\})\cap S_1\cap S_2 \end{aligned}$$

34 $$\begin{aligned}&(\mathbf{x},\mathbf{z})\in \{0,1\}^{|A|+|V|} \end{aligned}$$

To avoid the need of variables indicating the inclusion of node pairs, we use slightly modified connectivity constraints (31) and (32) ensuring that no nodes from the root cluster $V_r$ are contained in set *S*. Thus, the node selected within $V_r$ (one of them needs to be selected in any solution) is not contained in *S* as well and valid connectivity constraints are obtained by using the variable corresponding to a node to which (from which, respectively) a path may be realized on the right hand side of (31) and (32), respectively. The same argument is used for ensuring disjointness by (33) where neither $S_1$ nor $S_2$ may contain a node from $V_r$. Recall that due to (5) only arcs adjacent to two selected nodes may be used and thus, we eliminate infeasible solutions that would satisfy the connectivity constraints by using cheap edges out from $V_r$ (or into $V_r$, respectively) adjacent to (potentially different) non-selected nodes.

Theorem 3 shows that formulation $(\mathrm {C}_{u})$ is theoretically weaker than the conceptually similar flow formulation $(\mathrm {F}_u)$. For showing this result, let $\mathcal {P}(M)$ denote the polyhedron associated with the LP relaxation of some formulation *M* and $\mathrm {proj}_{\mathbf {x,z}}(\mathcal {P}(M))$ be its orthogonal projection to the $(\mathbf {x},\mathbf {z})$-space.

#### **Theorem 3**

Formulation $(\mathrm {F}_u)$ is stronger than formulation $(\mathrm {C}_{u})$, i.e., $\mathrm {proj}_{x,z}(\mathcal {P}(\mathrm {F}_u))\subseteq \mathcal {P}(\mathrm {C}_{u})$ and there exist instances for which the inclusion is strict.

#### *Proof*

**Fig. 2:**
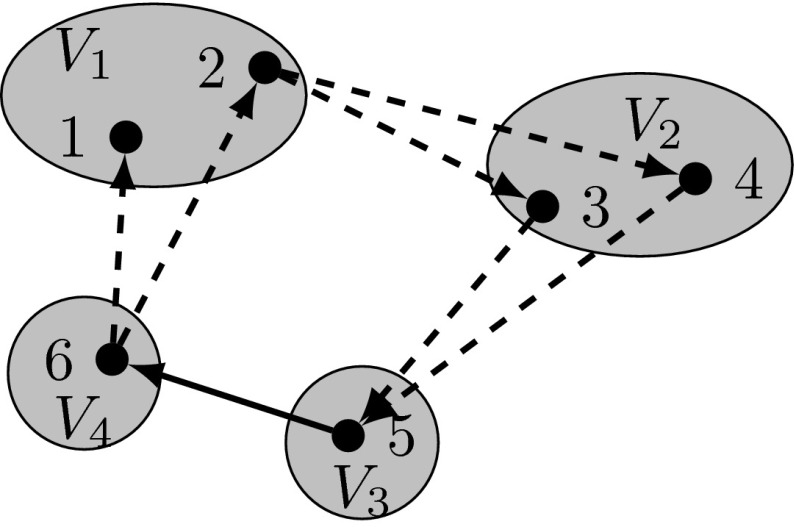
Graph of a feasible solution to $\mathcal {P}(\mathrm {C}_u)$ with $\rho _i=2, i=1,\ldots , 4, V_r=V_1, z_{5}=z_{6}=1, z_{i}=0.5, i=1,\ldots , 4, x_{56}=1, x_{uv}=0.5, (u,v)\in \{(2,3),(2,4),(3,5),(4,5),(6,1),(6,2)\}$

We first show that every solution in $\mathcal {P}(\mathrm {F}_u)$ satisfies constraints (31), (32), and (33), i.e., $\mathrm {proj}_{x,z}(\mathcal {P}(\mathrm {F}_u))\subseteq \mathcal {P}(\mathrm {C}_{u})$. Notice that despite the inequalities in flow conservation constraints (6) and (7) exactly $z_t$ units of flow $f^t$ ($g^t$) are sent out from (consumed by) the root cluster and consumed by (sent out from) node $t\in V(C_1\cup C_2\setminus \{r\})$ ($t\in V(C_2\setminus \{r\}$). Thus, from linking constraints (9), (10) and the max-flow-min-cut theorem, we conclude that every solution in $\mathcal {P}(\mathrm {F}_u)$ satisfies forward and backward cut constraints (31) and (32), respectively. Furthermore, from inequalities (8) we observe that for each $t\in V(C_2\setminus \{r\})$ at most $z_t$ units of the $2\cdot z_t$ units of flow $(f+g)^t$ are sent via arcs adjacent to any node $w\in V\setminus \{V_r\cup \{t\}\}$. Thus, at least $z_t$ units of flow $(f+g)^t$ are routed in $A\setminus A(w)$ and hence (33) follow from this observation, (9), (10), and the max-flow-min-cut theorem. To see that $(\mathrm {F}_u)$ is stronger than formulation $(\mathrm {C}_{u})$, consider the solution given in Fig. 2 which is feasible for $\mathcal {P}(\mathrm {C}_{u})$ but infeasible for $\mathcal {P}(\mathrm {F}_u)$ since flow conservation constraints (6) are violated, e.g. for $u=1$ and $t=5$. $\square$

Finally, we propose formulation $(\mathrm {CC})$ given by (35)–(40) that makes use of arc set $A_\mathrm{C}$ introduced in Sect. 2.1 to consider a solutions structure, i.e., its inter-cluster connections. Thereby, additional variables $y_{ij}\in \{0,1\}, \forall (i,j)\in A_\mathrm{C}$, will indicate whether or not an arc from a node in cluster *i* to a node in cluster *j* is included in the solution or not. Similar to model $(\mathrm {CF}), \delta _\mathrm {c}^+(S)$ ($\delta _\mathrm {c}^-(S)$) is used to refer to the outgoing (incoming) inter-cluster arcs from $A_\mathrm{C}$ for a set of clusters $S\subset C$.

35 $$\begin{aligned} \min \,&\sum _{(u,v)\in A} c_{uv} x_{uv} \end{aligned}$$

36 $$\begin{aligned} \text{ s.t. }\,&(2)-(5) \nonumber \\&x[\delta (V_i,V_j)] = y_{ij} \qquad (i,j)\in A_\mathrm{C} \end{aligned}$$

37 $$\begin{aligned}&y[\delta _\mathrm {c}^-(S)]\ge 1 \qquad S\subset C\setminus \{r\},\ S\cap (C_1\cup C_2\setminus \{r\})\ne \emptyset \end{aligned}$$

38 $$\begin{aligned}&y[\delta _\mathrm {c}^+(S)]\ge 1 \qquad S\subset C\setminus \{r\},\ S\cap (C_2\setminus \{r\})\ne \emptyset \end{aligned}$$

39 $$\begin{aligned}&y[\delta _\mathrm {c}^-(S_1)\setminus A_\mathrm{C}(w)] + y[\delta _\mathrm {c}^+(S_2)\setminus A_\mathrm{C}(w)] \ge 1 \qquad w\in C\setminus \{r\}, \nonumber \\&\ S_1, S_2\subset C\setminus \{r\},\ \exists j\in S_1\cap S_2\cap C_2, j\ne r \end{aligned}$$

40 $$\begin{aligned}&(\mathbf{x},\mathbf{y},\mathbf{z})\in \{0,1\}^{|A|+|A_\mathrm{C}+|V|} \end{aligned}$$

The objective function (35) as well as constraints (2)–(5) have been discussed before. Equation (36) are linking constraints ensuring that variable $y_{ij}$ is set to one whenever an arc from a node in cluster *i* to a node in cluster *j* is selected and also ensure that at most one such arc can be chosen. Inequalities (37) and (38) are the directed connectivity constraints appropriately modified to the arc set $A_\mathrm{C}$ (i.e., they enforce the existence of the required paths on an inter-cluster level). Node disjointness of the two paths associated to a cluster from $C_2$ is ensured by appropriately modified cutset constraints given by inequalities (39).

A stronger variant of $(\mathrm {CC})$ is obtained by additionally considering node cuts (31), (32), and (33). We will use $(\mathrm {CC})^+$ to refer to the variant augmented by these three sets of inequalities.

### Connection formulations

A third class of possible formulations is obtained from considering exponentially many variables rather than constraints. The basic idea is to introduce one variable for each feasible connection between the chosen root node and each other selected node from a mandatory cluster. Similar formulations which are typically solved by branch-and-price (i.e., by embedding column generation into branch-and-bound) have shown to yield quite effective solution methods for related problems, see, e.g. [25]. In the following we detail one such formulations that conceptually corresponds to $(\mathrm {C}_{u})$ and analyze the resulting pricing subproblems. In addition we describe necessary modifications to a further model that corresponds to $(\mathrm {CC})$.

To derive a set partitioning formulation, consider the set of feasible connections $\mathcal {P}_u\subseteq 2^{|A|}$ for each node $u\in V(C_1\cup C_2\setminus \{r\})$. For nodes $u\in V(C_1)$, set $\mathcal {P}_u$ contains all directed paths in (*V*, *A*) that start from a node $s\in V_r$ and end at node *u* which do contain at most one node from each cluster, i.e., $\mathcal {P}_u=\{\{(s=v_0,v_1), (v_1,v_2), \ldots , (v_{l-1},v_l=u)\} \mid s\in V_r, |V_i\cap (\bigcup _{j=0}^{l} v_j)|\le 1, 1\le i\le k, (v_j,v_{j+1})\in A, 0\le j < l\}$. Similarly, for nodes $u\in V(C_2\setminus \{r\})$, set $\mathcal {P}_u$ contains all directed cycles in (*V*, *A*) that contain nodes *u* and $s\in V_r$ and which do contain at most one node from each cluster, i.e., $\mathcal {P}_u=\{\{(s=v_0, v_1), (v_1,v_2), \ldots , (v_{l-1}, v_l=s)\}\mid s\in V_r, u\in \bigcup _{j=1}^{l-1} v_j, |V_i\cap (\bigcup _{j=0}^{l-1} v_j)|\le 1, 1\le i\le k, (v_j,v_{j+1})\in A, 0\le j < l\}$. Using variables $\lambda _p\in \{0,1\}, \forall p\in \bigcup _{u\in V(C_1\cup C_2\setminus \{r\})} \mathcal {P}_u$, indicating which feasible connections will be realized, formulation $(\mathcal {C}_u)$ is obtained from $(\mathrm {C}_{u})$ by replacing (31)–(33) by (41)–(43).

41 $$\begin{aligned}&\sum _{p\in \mathcal {P}_u} \lambda _p = z_u&u\in V(C_1\cup C_2\setminus \{r\}) \end{aligned}$$

42 $$\begin{aligned}&\sum _{p\in \mathcal {P}_u : (i,j)\in p} \lambda _p\le x_{ij}&u\in V(C_1\cup C_2\setminus \{r\}),\ (i,j)\in A \end{aligned}$$

43 $$\begin{aligned}&\lambda _p\in \{0,1\}&u\in V(C_1\cup C_2\setminus \{r\}), p\in \mathcal {P}_u \end{aligned}$$

Equations (41) ensure that one feasible connection is chosen for each node selected in a mandatory cluster while inequalities (42) are linking constraints ensuring that all arcs contained in at least one realized connection are included in the final solution. A valid formulation is obtained together with the objective function (30), the previously discussed constraints (2)–(5), and the definitional constraints (34) and (43).

*Pricing subproblem* To dynamically add connection variables, we need to identify $u\in V(C_1\cup C_2\setminus \{r\})$ and $p\in \mathcal {P}_u$ such that variable $\lambda _p$ has negative reduced costs or prove that no such variable exists. Associating dual variables $\mu _u$ and $\nu _{ij}^u\ge 0$ to constraints (41) and (42), respectively, a connection corresponding to a variable with minimum reduced can be identified by solving the optimization problem $\text{ argmin }_{u\in V(C_1\cup C_2\setminus \{r\}), p\in \mathcal {P}_u} \{ -\mu _u + \sum _{(i,j)\in p} \nu _{ij}^u \}$.

Thus, it suffices to find a cheapest feasible connection in (*V*, *A*) with respect to nonnegative arc costs $\nu _{ij}^u$ for each $u\in V(C_1\cup C_2\setminus \{r\})$. If the costs of such a connection are less than $\mu _u$ the corresponding variable has negative reduced costs. As we will, however, show by the following two theorems, identifying such a minimum cost connection is NP-hard both for nodes from type-1 and type-2 clusters.

#### **Theorem 4**

It is NP-hard to decide whether (*V*, *A*) contains a directed path from an arbitrary (but fixed) node in $V_r$ to a particular node $u\in V(C_1)$ that contains at most one node from each cluster $V_i, 1\le i\le k$.

#### *Proof*

This result follows by reduction from the path with forbidden pairs problem (PFPP). Given a directed graph $(\mathcal {V},\mathcal {A})$, two vertices $s,t\in \mathcal {V}$, and a collection $\mathcal {L}=(\{u_1,v_1\}, \ldots , \{u_n,v_n\})$, of pairs of vertices from $\mathcal {V}$, the PFPP is the problem of deciding whether there exists a directed path from *s* to *t* in $\mathcal {G}$ that contains at most one vertex from each pair in $\mathcal {L}$. The PFPP is NP-hard even if all forbidden pairs are disjoint [8]. A transformation of each such instance to the pricing subproblem for nodes in type-1 clusters is obtained by considering clusters $V_i=\{u_i,v_i\}, 1\le i\le n$, and $V_j=\{u_j\}, \forall u_j\in \mathcal {V}\setminus \bigcup _{i=1}^n V_i$ with $V_r=\{s\}$ and $u=t$. $\square$

The following result has been slightly rewritten but otherwise corresponds to an analogous one shown in Leitner et al. [18] via a reduction from the disjoint pair of paths problem. As it treats the special case when all clusters contain a single node only, it implies that the pricing subproblem for nodes $u\in V(C_2)$ is NP-hard as well.

#### **Theorem 5**

It is NP-hard to decide whether (*V*, *A*) contains a directed cycle including nodes $s\in V_r$ and $u\in V(C_2\setminus \{r\})$.

An alternative formulation conceptually corresponding to $(\mathrm {CC})$ is obtained from considering the set of feasible inter-cluster connections, i.e., directed paths (from the root cluster to the respective target cluster) and cycles (containing the root cluster and the respective target cluster) on the graph induced by $A_\mathrm{C}$. Considering the set of feasible inter-cluster connections $\mathcal {F}_u\subseteq 2^{|A_\mathrm{C}|}$ for each mandatory cluster $u\in C_1\cup C_2$ and associated decision variables $\pi _p\in \{0,1\}, \forall p\in \bigcup _{u\in C_1\cup C_2} \mathcal {F}_u$, a valid formulation is obtained from $(\mathrm {CC})$ by replacing (37)–(39) by (44)–(46).

44 $$\begin{aligned}&\sum _{p\in \mathcal {F}_u} \pi _p = 1&u\in C_1\cup C_2\setminus \{r\} \end{aligned}$$

45 $$\begin{aligned}&\sum _{p\in \mathcal {F}_u : (i,j)\in p} \pi _p\le y_{ij}&u\in C_1\cup C_2\setminus \{r\},\ (i,j)\in A_\mathrm{C} \end{aligned}$$

46 $$\begin{aligned}&\pi _p\in \{0,1\}&u\in C_1\cup C_2\setminus \{r\}, p\in \mathcal {F}_u \end{aligned}$$

The interpretation of these three sets of constraints is analogous to the one of (41)–(43). From the discussion above it is easy to conclude that for type-1 clusters $u\in C_1$, the pricing subproblem can be solved by computing a minimum cost path on the graph induced by $A_\mathrm{C}$ and given non-negative arc costs obtained from the dual multipliers associated to constraints (45). Similarly, for each $u\in C_2$, a minimum cost directed cycle containing the root cluster *r* and *u* needs to be identified. Thus, the pricing subproblem can be solved in polynomial time for type-1 clusters while it is NP-hard for type-2 clusters.

## Computational study

The flow formulations introduced in Sect. 2.1 as well as branch-and-cut algorithms corresponding to the models from Sect. 2.2 have been implemented in C++ using IBM CPLEX 12.6. In what follows, we do, however, not consider the first two models $(\mathrm {F}_{sv})$ and $(\mathrm {F}_{u})$ as preliminary experiments (as expected) showed their inferior performance due to the large numbers of variables involved and their weak LP relaxation bounds. We also refrain from considering the two connection formulations introduced in Sect. 2.3 since the computational results obtained in Leitner et al. [18] for a closely related problem are not very promising. In fact, the pricing subproblems arising in the present work even generalize the ones from Leitner et al. [18]. Hence, a good performance of the resulting branch-and-price approaches may only be possible through the use of sophisticated and clever pricing heuristics next to considering stabilization techniques and primal heuristics. Thus, we will compare the developed algorithms based on models $(\mathrm {CF}), (\mathrm {C}_{sv}), (\mathrm {C}_{u}), (\mathrm {CC})$, and $(\mathrm {CC})^+$.

An implementation of the push-relabel maximum flow algorithm by Cherkassky and Goldberg [1] has been used for separating the different classes of cutset constraints and we generally add cutset constraints only if they are violated by a value of at least 0.1 in the current LP solution. For $(\mathrm {C}_{sv})$ we only search for violated “disjointness cuts” (27) if no violated forward (25) or backward (26) cuts have been identified for the current solution. Strengthening inequalities (29) are initially added to the model. An analogous strategy is used for $(\mathrm {C}_{u}), (\mathrm {CC})$, and $(\mathrm {CC})^+$. For the latter, node cuts are only separated if no further cluster cuts are violated. Each computational experiment has been performed on a single core of a cluster of computers each consisting of 20 cores (2.3 GHz) and 64 GB RAM. An absolute time limit of 10,000 CPU-seconds and a memory limit of 2.5 GB has been applied to each individual run.

### Test instances

We created sets of benchmark instances with different relative percentage values of required and redundant clusters that are based on instances by Fischetti et al. [7] that have been widely used for the evaluation of algorithmic approaches to GNDPs, see, e.g. [13, 14]. Note that these original instances contain information about the underlying graph, edge costs and the assignment of nodes to clusters. Furthermore, for each original TSPlib [26] instance, five instances exist in which the assignment of nodes to clusters has been done either geographically or grid based (with different numbers of average nodes per cluster $\mu$ according to parameter $\mu \in \{3,5,7,10\}$), see [3, 7] for more details. The number of nodes of the underlying (complete) graph is encoded in the name of each instance, cf. Tables 1 and 2.

To create benchmark instances for the $\{0,1,2\}$-GSNDP, for each such instance we randomly select $\ell =\lceil r\cdot k\rceil , r\in \{0.5,0.75,1\}$, clusters to be mandatory and $\lceil r_2\cdot \ell \rceil , r_2\in \{0.5, 0.75, 1\}$, among those as redundant clusters. By repeating this process, five different instances have been created for each considered combination of *r* and $r_2$ except for the case $(r,r_2)=(1,1)$ where we would obtain five identical instances.

### Results

To analyze the performance of the branch-and-cut algorithms developed for the different models proposed in Sects. 2.1 and 2.2 we first discuss the results obtained for the five considered variants on instances with less than 100 nodes. Table 1 reports numbers of instances solved to proven optimality, average CPU-times in seconds, and average gaps ($\mathrm {gap}_\mathrm{root}$) in percent of the lower bound obtained from solving the root node of the branch-and-cut tree (or the current lower bound in case the root node could not be solved within the time- or memorylimit). These gaps are computed as $100\cdot (UB^*-LB)/UB^*$ where $UB^*$ is the value of the best known solution computed from any of the considered variants (i.e., the optimal cost in almost all cases) and *LB* is the root node lower bound of the respective variant. Notice that these gaps do not necessarily reflect the theoretical strength of the formulations due to presolving and preprocessing by CPLEX and since we only separate cutset constraints if they are violated by a value of at least 0.1. To gain insight into potential advantages and disadvantages of the methods, these results are grouped according to three different characteristics (original instance graph, clustering method, and relative amount of required and redundant clusters, respectively). The CPU-times of all experiments that terminated due to reaching the memory limit have been considered as 10,000 s when computing the average times.

**Table 1:** Numbers of instances solved to proven optimality ($\#_\mathrm{solved}$)

		#	$\#_\mathrm{solved}$					$t_\mathrm{avg}$ (s)					$\mathrm {Gap}_\mathrm{root} (\%)$				
			$(\mathrm {CF})$	$(\mathrm {C}_{sv})$	$(\mathrm {C}_{u})$	$(\mathrm {CC})$	$(\mathrm {CC})^+$	$(\mathrm {CF})$	$(\mathrm {C}_{sv})$	$(\mathrm {C}_{u})$	$(\mathrm {CC})$	$(\mathrm {CC})^+$	$(\mathrm {CF})$	$(\mathrm {C}_{sv})$	$(\mathrm {C}_{u})$	$(\mathrm {CC})$	$(\mathrm {CC})^+$
Inst	att48	205	**205**	**205**	**205**	204	**205**	138	47	13	59	**8**	17.1	59.5	23.3	17.0	**10.3**
	eil51	205	**205**	**205**	**205**	**205**	**205**	493	31	8	12	**7**	14.1	63.3	13.6	14.3	**9.4**
	st70	205	192	**205**	**205**	181	**205**	1497	250	32	1219	**25**	17.4	74.8	17.6	17.6	**10.2**
	eil76	205	141	**205**	203	103	201	4501	471	**162**	5034	255	21.4	66.5	18.7	21.5	**13.0**
	pr76	205	135	203	**205**	65	**205**	5257	447	73	6886	**71**	24.3	51.2	15.0	24.3	**10.3**
	gr96	205	92	194	**203**	30	201	7466	1650	**313**	8560	374	17.9	67.0	9.9	17.8	**6.6**
	rat99	205	11	110	200	1	**202**	9778	5645	942	9953	**699**	28.4	79.6	27.1	29.3	**20.7**
Clust	geo	287	186	267	**287**	123	286	4672	1175	**221**	5739	229	22.8	67.8	21.0	22.9	**13.5**
	$\mu =3$	287	113	**287**	284	129	281	6886	**159**	166	5531	259	9.5	41.9	3.9	10.2	**1.9**
	$\mu =5$	287	212	282	283	160	**284**	3662	831	253	4451	**197**	16.9	64.7	14.4	17.2	**8.7**
	$\mu =7$	287	233	245	286	165	**287**	3279	1821	253	4281	**173**	23.8	75.0	21.8	23.9	**14.0**
	$\mu =10$	287	237	246	**286**	212	**286**	2308	2115	209	2658	**169**	27.5	80.5	28.4	27.2	**19.4**
$(r,r_2)$	(0.5,0.5)	175	123	161	**175**	113	**175**	3560	1361	208	3589	**154**	22.3	65.0	23.6	23.1	**13.7**
	(0.5,0.75)	175	126	163	**175**	106	**175**	3790	1131	133	3984	**109**	22.0	67.1	20.5	23.1	**12.9**
	(0.5,1)	175	129	165	**175**	105	**175**	3478	865	108	4030	**92**	21.4	64.7	19.7	22.3	**12.3**
	(0.75,0.5)	175	117	158	173	97	**174**	4218	1548	337	4495	**232**	20.4	63.7	17.5	20.4	**11.7**
	(0.75,0.75)	175	115	163	**175**	90	**175**	4409	1113	170	4881	**146**	20.3	66.7	16.6	20.2	**11.4**
	(0.75,1)	175	114	163	**175**	88	173	4413	1046	**109**	4989	205	19.2	66.1	15.7	18.8	**10.3**
	(1,0.5)	175	116	157	171	89	**173**	4441	1563	384	4940	**263**	18.2	66.1	15.6	17.7	**10.3**
	(1,0.75)	175	117	164	**172**	84	170	4852	1177	**319**	5220	400	17.8	67.8	15.0	17.3	**9.8**
	(1,1)	35	24	33	**35**	17	34	4820	1010	**197**	5164	421	17.3	69.3	13.1	16.2	**9.0**
Total	-	1435	981	1327	**1426**	789	1424	4162	1220	220	4532	**206**	20.1	66.0	17.9	20.3	**11.5**

Best values are given in bold

Average CPU-times in seconds ($t_\mathrm{avg}$), and average gaps after solving the root node in percent ($\mathrm {gap}_\mathrm{root}$) of branch-and-cut algorithms based on different models grouped by original instance (Inst), clustering method (Clust), and relative amounts of required and redundant clusters $(r,r_2)$, respectively. Average CPU-times (rounded to the nearest integer) have been computed using a value of 10,000 s whenever an approach terminated earlier due to the memory limit

We observe that, even though the number of flow variables of $(\mathrm {CF})$ is significantly smaller than for the other flow models it is only able to solve approximately two-thirds of the instances. Its performance (relative to the other variants) clearly improves when the number of clusters is relatively small compared to the number of nodes (i.e., for instances with grid clustering and $\mu \in \{7,10\}$). Given the fact that its implementation requires significantly less effort compared to the variants with an exponential number of dynamically added inequalities, it might therefore be a viable option for medium sized instances with a moderate number of clusters. Surprisingly, variant $(\mathrm {CC})$ which is also based on the idea of focusing on inter-cluster connection, but models them by means of dynamically separated directed cutset constraints performs even worse than $(\mathrm {CF})$. Notice that the observed root node gaps also indicate that the lower bounds at the root node from these two variants are almost identical. We suppose that one reason for the higher efficiency of $(\mathrm {CF})$ might be a better performance of the general purpose heuristics implemented in CPLEX for compact models where complete information is available to the solver. In addition the presolving, probing, and bound strengthening routines of CPLEX as well as identification of general purpose valid inequalities may be more effective for the same reason.

It is also rather surprising that $(\mathrm {C}_{uv})$ involving the “quadratic” variables denoting whether pairs of nodes are selected, outperforms $(\mathrm {CC})$ and $(\mathrm {CF})$ in particular since its root node gaps are extremely large (in accordance with Theorem 2). We conclude, that a large number of nodes from the branch-and-cut tree that can be processed relatively fast seems to partly compensate the poor bounds (obtained in the root node). We also observe that the performance $(\mathrm {C}_{uv})$ deteriorates with an increasing number of average nodes per cluster (i.e., with increasing value of $\mu$). Clearly, a smaller number of nodes per cluster (and thus much less choices for node pairs in the solution) will typically lead to better bounds already after few branching decisions given the chosen linearization of the quadratic variables.

Finally, we observe that the branch-and-cut based on cuts to individual nodes $(\mathrm {C}_{u})$ and variant $(\mathrm {CC})^+$ combining cluster and node cuts clearly outperform the other options. Slight advantages with respect to the total number of solved instances and for cases with relatively few numbers of nodes per cluster can be observed for $(\mathrm {C}_{u})$ while the root node gaps of $(\mathrm {CC})^+$ are consistently smaller than the one of $(\mathrm {C}_{u})$. In general, however, their performance does not differ too much on the instances considered in Table 1.

To gain additional insights on the relative performance of these two variants an additional set of results on larger instances containing at least 100 nodes is given in Table 2. Besides the numbers of instances solved to proven optimality, average CPU-times (again all cases of terminations due to the memory limit have been considered as 10,000 s) and average root node gaps in percent, we also report the numbers of cases in which one of the two algorithms outperformed the other. Thereby, an algorithm is considered to outperform the other on an instance, if it solved it to proven optimality faster (with a difference of at least 10 s) or alternatively if its optimality gap is smaller (by at least 1 %) in case none of the two variants solved the instance to proven optimality.

**Table 2:** Numbers of instances solved to proven optimality ($\#_\mathrm{solved}$)

		#	$\#_\mathrm {solved}$		$t_\mathrm {avg}$ (s)		$\#_\mathrm {best}$		$\mathrm {Gap}_\mathrm{root} (\%)$	
			$(\mathrm {C}_{u})$	$(\mathrm {CC})^+$	$(\mathrm {C}_{u})$	$(\mathrm {CC})^+$	$(\mathrm {C}_{u})$	$(\mathrm {CC})^+$	$(\mathrm {C}_{u})$	$(\mathrm {CC})^+$
Inst	kroa100	205	182	**201**	1630	**692**	56	**138**	21.5	**15.3**
	krob100	205	168	**184**	2327	**1442**	39	**149**	21.2	**14.0**
	kroc100	205	162	**178**	2635	**1709**	29	**159**	24.7	**18.1**
	krod100	205	184	**192**	1516	**1023**	55	**136**	18.8	**12.9**
	kroe100	205	191	**202**	1048	**401**	31	**153**	14.8	**8.7**
	rd100	205	190	**192**	1019	**822**	38	**140**	16.5	**10.7**
	eil101	205	199	**201**	649	**543**	91	**97**	20.1	**13.8**
	pr107	205	117	**199**	4998	**681**	0	**205**	36.6	**4.6**
	pr124	205	92	**143**	5893	**3361**	10	**185**	25.7	**14.8**
	bier127	205	106	**108**	5082	**4990**	72	**97**	13.8	**11.4**
Clust	geo	410	308	**355**	3086	**1851**	107	**275**	24.7	**13.8**
	$\mu =3$	410	294	**310**	2986	**2544**	86	**265**	11.7	**9.9**
	$\mu =5$	410	337	**370**	2123	**1183**	69	**299**	15.1	**6.4**
	$\mu =7$	410	327	**378**	2603	**1237**	72	**310**	26.6	**15.6**
	$\mu =10$	410	325	**387**	2601	**1017**	87	**310**	28.8	**16.4**
$(r,r_2)$	(0.5,0.5)	250	225	**240**	1649	**825**	51	**183**	25.1	**12.4**
	(0.5,0.75)	250	226	**243**	1424	**624**	52	**168**	24.6	**12.6**
	(0.5,1)	250	233	**240**	1087	**648**	51	**170**	21.3	**11.6**
	(0.75,0.5)	250	188	**220**	2987	**1662**	54	**177**	19.9	**11.3**
	(0.75,0.75)	250	189	**220**	2847	**1532**	46	**189**	19.6	**11.3**
	(0.75,1)	250	187	**213**	2859	**1746**	50	**177**	20.1	**12.3**
	(1,0.5)	250	155	**198**	4201	**2476**	51	**190**	19.7	**12.7**
	(1,0.75)	250	160	**191**	3969	**2682**	57	**170**	19.9	**13.6**
	(1,1)	50	28	**35**	4753	**3247**	9	**35**	26.0	**20.3**
Total	-	2050	1591	**1800**	2680	**1566**	421	**1459**	21.4	**12.4**

Best values are given in bold

Average CPU-times in seconds ($t_\mathrm{avg}$), numbers of cases where an approach obtained the best performance ($\#_\mathrm{best}$), and average gaps after solving the root node in percent ($\mathrm {gap}_\mathrm{root}$) for $(\mathrm {C}_u)$ and $(\mathrm {CC})^+$ grouped by original instance, clustering method, and relative amounts of required and redundant clusters, respectively. Average CPU-times (rounded to the nearest integer) have been computed using a value of 10,000 s whenever an approach terminated earlier due to the memory limit. An algorithm is considered to yield a better performance than another one, if it could solve an instance to proven optimality at least 10 s faster, if the other one could not solve the corresponding instance, or if the remaining optimality gap was at least 1 % smaller in case both algorithms failed to solve the instance

The results from Table 2 show that both $(\mathrm {C}_{u})$ and $(\mathrm {CC})^+$ perform reasonably good on the considered set of larger instances. Despite the fact the differences between the two variants are not too large in some cases one can clearly observe that $(\mathrm {CC})^+$ outperforms $(\mathrm {C}_{u})$ with respect to all considered criteria. No clear correlation between their relative performance and the size of the underlying instances can be observed. To this end, we note that the performance of $(\mathrm {C}_{u})$ and $(\mathrm {CC})^+$ is almost identical for the largest instances considered (i.e, those based on instance bier127). More conclusions can be drawn when considering the average number of nodes per cluster, i.e., the influence of parameter $\mu$. Despite an increasing root node gap, the efficiency of $(\mathrm {CC})^+$ clearly improves with an increasing value of $\mu$ (more instances can be solved to optimality and the average CPU-times tend to decrease). On the other hand, $(\mathrm {C}_{u})$ exhibits a relatively stable (but significantly worse) performance independent of the average number of nodes per cluster. We also conclude (for both variants) that the difficulty of an instance seems to correlate with the number of required clusters while this is not so clear for the relative number of redundant clusters. Independently of the considered combination $(r, r_2)$, however, $(\mathrm {CC})^+$ outperforms $(\mathrm {C}_{u})$. While their relative difference is relatively constant among the considered combinations with respect to average CPU-times and numbers of cases where one of the two achieves a better performance, it tends to slightly increase with respect to the numbers of solved instances with increasing value of *r* and/or $r_2$.

Overall, we conclude that both $(\mathrm {C}_{u})$ and $(\mathrm {CC})^+$ achieve a relatively good and stable performance with clear advantages of the latter variant which seems particularly well suited for instances with many nodes per cluster and many mandatory or redundant clusters.

## Conclusions

In this article, we studied the Generalized $\{0,1,2\}$-Survivable Network Design Problem a new survivable network design problem that arises in the context of backbone network design and generalizes well-known GNDPs as well as classical problems from survivable network design. Using a recent orientation result with respect to two-node connected graphs by Chimani et al. [2], a number of MILP formulations based on multi-commodity flows, directed cutset constraints, and exponentially many connection variables have been derived. One aim of the article was derive formulations with less variables/constraints by focusing on inter-cluster connections, a concept that is known to be quite effective for related generalized network design problems. Our computational study on a large set of benchmark instances revealed that the achieved reduction of the formulation size is partly foiled by weaker LP formulations. It also turned out, however, that combining this formulation with standard cutset constraints yields a variant that clearly outperforms all other variants studied in this article. The latter is particularly true when the number of nodes per cluster is relatively large.

Aspects that could be considered in future research include the development of branch-and-price approaches based on the connection formulations introduced in Sect. 2.3. To this end, primal and pricing heuristics as well as stabilization techniques and careful tuning of parameters are likely to be necessary in order to obtain a good performance.
